# Transformation and scattering activities of the receptor tyrosine kinase RON/Stk in rodent fibroblasts and lack of regulation by the jaagsiekte sheep retrovirus receptor, Hyal2

**DOI:** 10.1186/1471-2407-4-64

**Published:** 2004-09-13

**Authors:** A Dusty Miller, Neal S Van Hoeven, Shan-Lu Liu

**Affiliations:** 1 Division of Human Biology, Fred Hutchinson Cancer Research Center, 1100 Fairview Avenue North, Seattle, WA 98109-1024, USA; 2 Molecular and Cellular Biology Program, Fred Hutchinson Cancer Research Center, 1100 Fairview Avenue North, Seattle, WA 98109-1024, USA

## Abstract

**Background:**

The envelope (Env) protein of jaagsiekte sheep retrovirus (JSRV) can transform cells in culture and is likely to be the main factor responsible for lung cancer induction by JSRV in animals. A recent report indicates that the epithelial-cell transforming activity of JSRV Env depends on activation of the cell-surface receptor tyrosine kinase Mst1r (called RON for the human and Stk for the rodent orthologs). In the immortalized line of human epithelial cells used (BEAS-2B cells), the virus receptor Hyal2 was found to bind to and suppress the activity of RON. When Env was expressed it bound to Hyal2 causing its degradation, release of RON activity from Hyal2 suppression, and activation of pathways resulting in cell transformation.

**Methods:**

Due to difficulty with reproducibility of the transformation assay in BEAS-2B cells, we have used more tractable rodent fibroblast models to further study Hyal2 modulation of RON/Stk transforming activity and potential effects of Hyal2 on RON/Stk activation by its natural ligand, macrophage stimulating protein (MSP).

**Results:**

We did not detect transformation of NIH 3T3 cells by plasmids expressing RON or Stk, but did detect transformation of 208F rat fibroblasts by these plasmids at a very low rate. We were able to isolate 208F cell clones that expressed RON or Stk and that showed changes in morphology indicative of transformation. The parental 208F cells did not respond to MSP but 208F cells expressing RON or Stk showed obvious increases in scattering/transformation in response to MSP. Human Hyal2 had no effect on the basal or MSP-induced phenotypes of RON-expressing 208F cells, and human, mouse or rat Hyal2 had no effect on the basal or MSP-induced phenotypes of Stk-expressing 208F cells.

**Conclusions:**

We have shown that RON or Stk expression in 208F rat fibroblasts results in a transformed phenotype that is enhanced by addition of the natural ligand for these proteins, MSP. Hyal2 does not directly modulate the basal or MSP-induced RON/Stk activity, although it is possible that adaptor proteins might mediate such signaling in other cell types.

## Background

JSRV is an acutely oncogenic retrovirus that can induce lung tumors in newborn sheep in as little as 10 days [1]. Unlike most acutely oncogenic retroviruses, JSRV does not carry a host cell-derived oncogene. Instead, expression of the native viral Env protein is sufficient to transform cultured cells and is likely to be the active oncogene in animals [2-5]. The only other examples of such retroviruses are enzootic nasal tumor virus, a close relative of JSRV that encodes a similar transforming Env protein [6,7]; avian hemangioma virus [8], which expresses an Env protein that can induce cell proliferation in cultured cells [9]; and spleen focus-forming virus, a replication-defective virus that expresses a recombinant nonfunctional Env protein that has been shown to induce proliferation in cultured cells and in animals by activating the erythropoietin receptor and a short form of the transmembrane receptor tyrosine kinase Stk [10,11].

At least two mechanisms of transformation by JSRV Env have been identified in cultured cells. JSRV Env can transform immortalized mouse, rat, and chicken fibroblasts [2-4], and the cytoplasmic tail of Env is absolutely required for transformation [4,12-14]. Interaction of the extracellular domain of Env with the virus receptor Hyal2 appears to play no role in transformation of the rodent fibroblasts since JSRV Env does not bind to mouse Hyal2 [15], and deletion of the Hyal2-binding domain of Env did not abrogate transformation of rat fibroblasts [16]. Although there is some controversy regarding the mechanism of transformation, most studies indicate a role for the phosphatidylinositol 3-kinase (PI3K)/Akt pathway in transformation of the rodent fibroblasts [7,12,13,17]. In contrast, this pathway appears to play a smaller role in the transformation of chicken fibroblasts, but other more important pathways have yet to be identified [4,14].

A second pathway for JSRV Env transformation involves activation of the transmembrane receptor tyrosine kinase Mst1r (herein called RON for the human and Stk for the rodent forms [18,19]). RON/Stk activation is associated with cell scattering, cell transformation, and oncogenesis in animals [20]. RON/Stk activity is induced by binding of its only known ligand, macrophage stimulating protein (MSP) [21,22]. Recent evidence indicates that the JSRV receptor Hyal2 is also involved in regulating RON activity in an immortal human epithelial cell line (BEAS-2B) that naturally expresses Hyal2 and RON [5]. In its normal inactive state, RON is bound to Hyal2, and when Hyal2 binding is prevented by JSRV Env, RON becomes constitutively active in the absence of MSP stimulation. Introduction of a dominant negative form of RON into these cells blocked transformation, indicating that main transformation pathway is through RON and that JSRV might cause cancer through a novel mechanism involving RON activation [5]. Thus it appears that JSRV Env can transform cells by at least two distinct mechanisms. Furthermore, the dependence of epithelial cell transformation on activation of the RON pathway, in contrast to fibroblasts, which do not express RON and are transformed through a different pathway, indicated that the RON pathway might be more important in oncogenesis in animals because epithelial cells are the natural target for JSRV-induced cancer in sheep.

We have had difficulty in further exploring transformation through the RON pathway in BEAS-2B cells due to difficulty with reproducibility of the transformation assay. Others have found that Stk can transform immortalized rodent fibroblasts [23,24], and we hypothesized that if Hyal2 does downregulate RON/Stk activity, we might be able to suppress transformation by RON/Stk by overexpressing Hyal2. In addition, we wanted to explore whether Hyal2 might modulate MSP induction of RON/Stk activity. Here, we have been able to generate 208F rat fibroblasts that express either RON or Stk and find that these cells exhibit a transformed phenotype in comparison to the parental cells. Treatment of the cells with MSP induced a dose-dependent increase in scattering/transformation. Using a variety of approaches we have been unable to detect an effect of Hyal2 on the constitutive or MSP-induced phenotypes of 208F cells expressing RON or Stk. These results argue against a direct role for Hyal2 in the regulation of RON/Stk activity.

## Methods

### Cell culture

Cell lines used here include 208F Fischer rat embryo fibroblasts [25], a morphologically flat subclone of NIH 3T3 Swiss mouse embryo fibroblasts suitable for transformation studies (gift from Maxine Linial, Fred Hutchinson Cancer Research Center, Seattle; originally from Doug Lowy, National Cancer Institute, Bethesda), PJ4/LAPSN packaging cells [26] that produce the LAPSN retroviral vector [27] with a JSRV pseudotype, and PT67 10A1-MLV-pseudotype retrovirus packaging cells [28]. Unless otherwise stated, cells were grown in Dulbecco's modified Eagle medium (DMEM) with high glucose (4.5 g/L) and 10% fetal bovine serum at 37°C in a 10% CO_2_-air atmosphere at 100% relative humidity.

### Expression plasmids

Mouse Stk was expressed by using a plasmid (gift from Sandra Ruscetti, National Cancer Institute, Frederick, Maryland) that contains the mouse Stk cDNA cloned into the *Not*I site of the pAlter-Max expression plasmid (Promega, Madison, Wisconsin). Human RON was expressed by using a plasmid (gift from Michael Lerman, National Cancer Institute, Frederick, Maryland) that contains the human RON cDNA inserted into the pCI-neo expression plasmid (Promega). The expression cassette in both of these plasmids is identical and consists of a human cytomegalovirus immediate early promoter followed by splicing signals, the cDNA, and the simian virus 40 late polyadenylation signal. Mouse Stk was also expressed using the previously described pcDNA3-based expression vector [23] (gift from Susan Waltz, University of Cincinnati, Ohio), which contains the human cytomegalovirus early promoter, no splice signals, and the bovine growth hormone polyadenylation signal. Human RON was also expressed using the previously described retroviral vector expression plasmid pMSCVpuroRON [5], in which the RON cDNA is expressed from the Moloney murine leukemia virus promoter and the puromycin resistance gene is expressed using a phosphoglycerate kinase promoter. In this vector both spliced and unspliced mRNAs are made that encode RON.

### Retroviral vectors

The LAPSN retroviral vector encodes human placental alkaline phosphatase (AP) and neomycin phosphotransferase (Neo). LAPSN virus was made using PT67 (10A1-MLV pseudotype) or PJ4 (JSRV pseudotype) packaging cells. Retroviral vectors encoding human Hyal1, human Hyal2, mouse Hyal2 cloned from NIH 3T3 cells, and rat Hyal2 cloned from 208F rat cells (GenBank accession numbers U03056, U09577, AF535140, and AF535141, respectively) were made by insertion of the cDNAs into the LXSN vector [29] and by generation of stable PT67 retrovirus packaging cell lines that produce the vectors as described [3,15,29]. The human Hyal1 and human Hyal2 vectors present in the packaging cell clones that were used are known to be functional based on phenotypic assays of the proteins made in cells transduced with the vectors [3]. Two independent packaging cell clones that produce the rat Hyal2 vector and two independent clones that produce the mouse Hyal2 vector were used, and the correct sequence of the cDNAs in the integrated vectors in these packaging cells was confirmed by PCR amplification of the sequences followed by complete sequencing of the cDNAs.

### Cell transformation assay

NIH 3T3 and 208F cells were transfected using calcium phosphate as previously described [15] and were monitored for appearance of transformed foci of cells for up to a month after transfection.

### Cellular response to MSP

Recombinant human MSP activated by treatment with kallikrein (R&D Systems, Minneapolis, MN) was dissolved at a concentration of 10 μg/ml in phosphate buffered saline with calcium and magnesium containing 0.2% bovine serum albumin and was stored at -70°C. Cells were seeded at 10,000 per well (d = 16 mm) of 24-well plates. Two days after seeding, the cells were treated with 0.06 to 20 μl of MSP stock in 0.5 ml of fresh medium (MSP concentration of 1.2 to 400 ng/ml) and cellular morphology was evaluated the day after MSP addition. Treatment of cells with 20 μl buffer containing 0.2% bovine serum albumin in 0.5 ml of fresh medium had no effect on cell morphology.

## Results

### RON and Stk exhibit low transforming activity in rodent fibroblasts

We tested plasmids encoding RON or Stk (pCIneoRON and pAlter-Max-Stk, respectively) for transforming activity in NIH 3T3 mouse and 208F rat fibroblasts. The cDNAs in both plasmids were driven by a strong human cytomegalovirus immediate early promoter and each contained an intron to promote high-level expression. In these assays, strong oncogenes induce transformed foci of cells in less than a week while less active oncogenes take longer to induce foci. In both cases the numbers of foci generally increase with time after transfection.

Neither the RON nor the Stk expression plasmid induced transformed foci in NIH 3T3 cells (<0.2 foci per μg plasmid DNA at one month after transfection), while a plasmid expressing JSRV Env (pSX2.Jenv) induced 33 well-developed foci per μg plasmid DNA under the same conditions. This result is consistent with previous reports showing a lack of transformation by human RON [30-32], but is inconsistent with reports indicating efficient transformation of NIH 3T3 cells by mouse Stk (called "Ron" in these reports) [23,24]. We obtained the Stk expression plasmid used by the latter group (full-length mouse Ron cDNA in pcDNA3), and again could not detect transformation of NIH 3T3 cells by this plasmid (<0.2 foci per μg plasmid DNA at one month after transfection). There was some background focal growth in all plates of transfected NIH 3T3 cells one month after transfection, but this growth was identical in plates transfected with RON/Stk or control expression plasmids. In contrast, foci induced by JSRV Env were distinct and readily detected at 2 weeks after transfection.

Next we tested for transformation of 208F rat fibroblasts by the RON and Stk plasmids. The Stk plasmid (pAlter-Max-Stk) induced 3 clear foci in a total of 7 dishes (0.2 foci per μg plasmid DNA), and the RON plasmid (pCIneoRON) induced 1 clear focus in a total of 5 dishes (0.1 foci per μg plasmid DNA) (foci counted 1 month after transfection, 2 μg plasmid DNA per dish, 2–3 separate experiments for each plasmid). The Stk expression plasmid pcDNA3Stk used in the earlier studies [23,24] did not induce transformed foci in the 208F cells (<0.1 foci per μg plasmid DNA). For comparison, the JSRV Env expression plasmid induced 300 to over 500 foci per dish when counted 2 weeks after transfection under the same conditions. Together these results document a very low transforming activity for the RON and Stk genes.

### Generation of cells expressing RON and Stk that respond to MSP

Foci of transformed 208F cells were isolated from plates of cells transfected with the RON or Stk expression plasmids and were tested for their response to MSP. Cells transformed by the RON plasmid were not responsive to MSP and were discarded. Cells transformed by the Stk plasmid were responsive to MSP, and two clones (208F/Stk c12 and c13) were used for further study. Both of these clones made Stk protein of the appropriate molecular weight as judged by western analysis using RON/Stk-specific antibodies (data not shown).

**Figure 1:**
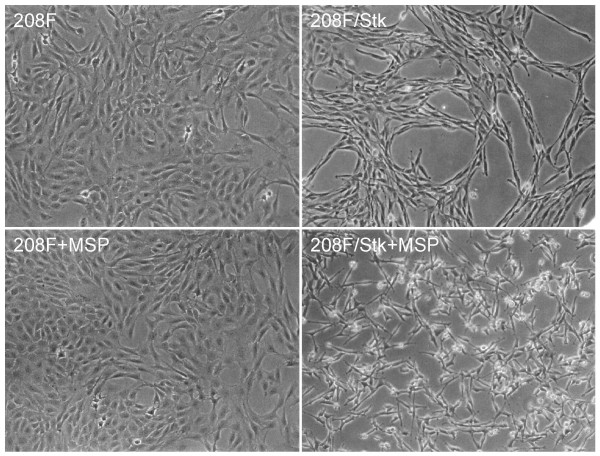
Scattering of 208F/Stk cells in response to MSP. 208F (left panels) and 208F/Stk c13 cells (right panels) were seeded at 2 × 10^4 ^cells per well in 24-well plates. Two days later the cells were treated overnight with 400 ng/ml MSP in growth medium (DMEM plus 10% FBS) (bottom panels) or with medium alone (top panels) and were photographed.

The response of 208F/Stk c13 cells to overnight treatment with MSP is shown in Fig. 1. Without MSP the cells were elongated and formed chains of connected cells (Fig. 1, top right panel), in contrast to the parental 208F cells which grow as flat uniformly-expanding colonies (Fig. 1, top left panel). Treatment of the 208F/Stk c13 cells with MSP resulted in dramatic scattering (Fig. 1, bottom right panel) while treatment of the parental 208F cells with MSP had no effect on cell morphology (Fig. 1, bottom left panel). The MSP response was fully reversible; incubation of cells overnight in regular growth medium reversed the scattering induced by MSP and chains of cells reformed (not shown). 208F/Stk c12 cells exhibited an even more transformed appearance than did 208F/Stk c13 cells, and many of these cells would grow in suspension, often as doublets of what appeared to be recently divided cells. These floating cells could be repeatedly regrown in new plates, showing that the cells were alive and were not simply undergoing apoptosis. Passage of the floating cells provided a simple method for maintaining the transformed phenotype of these cells, which tended to decrease with time of culture.

Since we were unsuccessful in generating 208F cells expressing functional RON by isolation of transformed cells following transfection, we isolated RON-expressing cells by transfection with a RON expression vector that also expressed a selectable marker (pMSCVpuroRON), grew the cells in puromycin to select for cells expressing the marker, and isolated multiple cell clones that showed a weakly-transformed phenotype. These clonal lines were screened for scattering response to MSP, and two independent clones that had the most transformed appearance and that responded to MSP (208F/RON c9 and c10) were identified and used in further analyses. Both clones produced RON protein of the appropriate size band by western analysis using a RON/Stk-specific antibodies, and both exhibited clear surface expression of RON by FACS analysis using a RON-specific antibody while control 208F cells showed no expression (data not shown). The 208F/RON clones had a less obvious transformed phenotype than the 208F/Stk clones but still showed convincing scattering in response to MSP (see below).

### Hyal2 expression does not affect the phenotype of cells expressing RON or Stk

**Figure 2:**
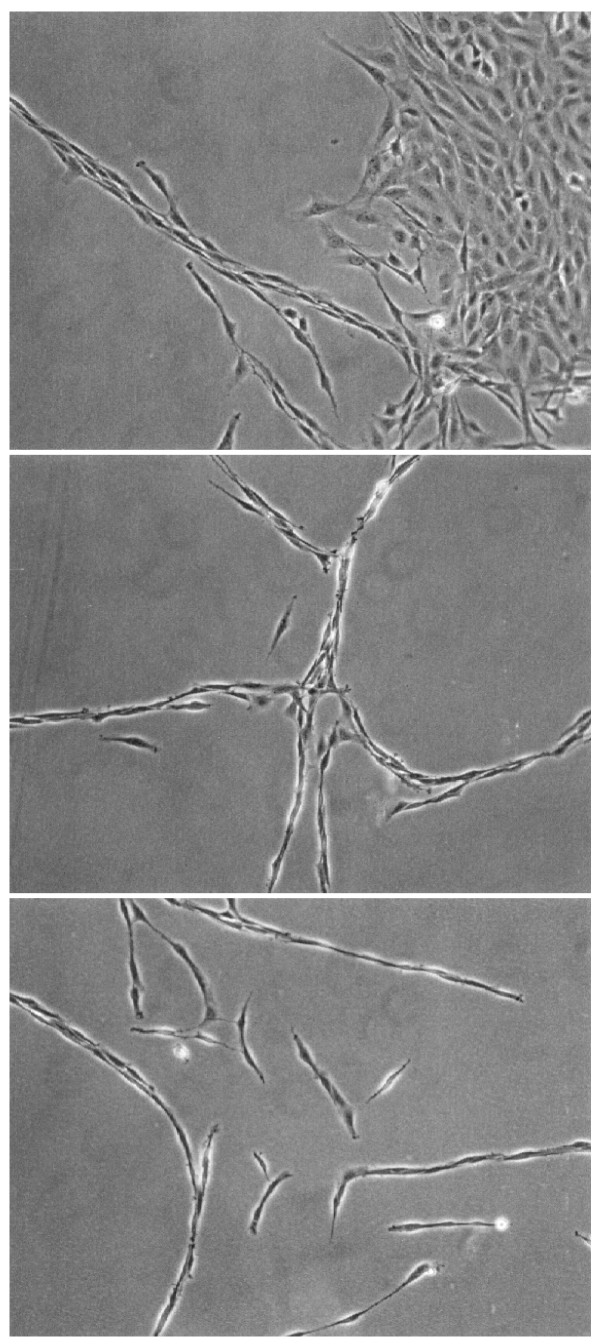
Morphology of 208F/Stk colonies. Cells were seeded 5 days before photographs were taken. See text for descriptions.

We tested whether overexpression of Hyal2 would reverse the altered phenotype of 208F cells expressing RON or Stk. Since the original work in BEAS-2B human cells showed inhibition of RON activity by human Hyal2 [5], we tested human Hyal2 in the 208F/RON cells. In 208F/Stk cells we tested mouse and rat Hyal2, as well as human Hyal2, in case the rodent proteins might show more effective interaction with mouse Stk in the rat cells. To quantitate the effect of Hyal2 expression on cell phenotype, cells were seeded at low density and colonies that grew out were scored for morphology, either round and flat, round and refractile, chain-like, or scattered. Fig. 2 shows examples of the phenotypes exhibited by 208F/Stk c13 cells. The top panel shows examples of chain-like (left) and flat (right) colonies, the middle panel shows an example of a chain-like colony, and the bottom panel shows an example of a scattered colony. In initial experiments we found that transduction of the cells with control vectors encoding either AP or Hyal1 had no effect on cell morphology (data not shown), and we used these vectors as negative controls for the effects of vector transduction in subsequent experiments.

**Figure 3:**
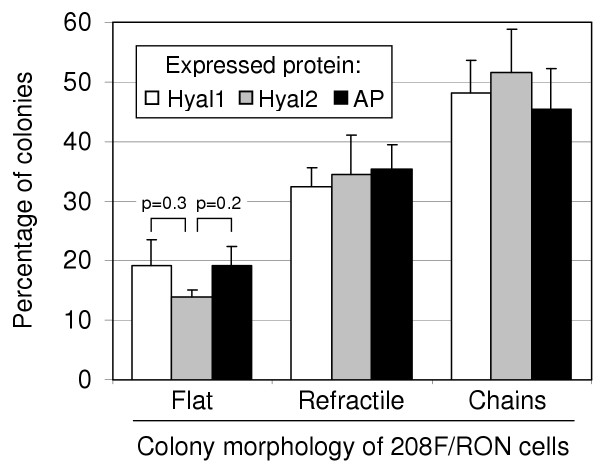
Colony morphology of 208F/RON cells is unaffected by Hyal2 expression. 208F/RON c9 and c10 cells were transduced with retrovirus vectors expressing the indicated proteins, the cells were seeded at low density, and colony morphologies were evaluated 1 week after seeding. Results are means of four experiments (two for each clone) and standard errors are shown.

We exposed 208F cells that expressed RON to retroviral vectors encoding human Hyal2, AP, or human Hyal1, grew these cells in G418 to select for cells expressing the vector, and quantitated the morphologies of the cells that grew out. The experiment was repeated by replating the G418-resistant cells at low density and repeating the morphologic quantitation. The results were similar for both cell lines in both experiments so the results for the two experiments with each of the two cell lines were averaged (Fig. 3). There was no significant change in morphology of the cells expressing human Hyal2 in comparison to those expressing human Hyal1 or AP, arguing against the hypothesis that Hyal2 can inhibit the activity of RON. Indeed, cells expressing human Hyal2 exhibited fewer flat colonies and more chain-like colonies than the other cell types, the opposite of what the hypothesis predicts, although these differences were not statistically significant (Fig. 3). To confirm that Hyal2 was expressed in these cells, we measured transduction of the cells by a JSRV-pseudotype retroviral vector, which cannot infect rat cells unless they express a functional Hyal2 receptor [15]. We confirmed that only the 208F/RON cells transduced with the human Hyal2 vector, and not those transduced with the human Hyal1 vector, were susceptible to JSRV-pseudotype LAPSN vector transduction, showing that functional human Hyal2 was indeed expressed in these cells (data not shown).

**Figure 4:**
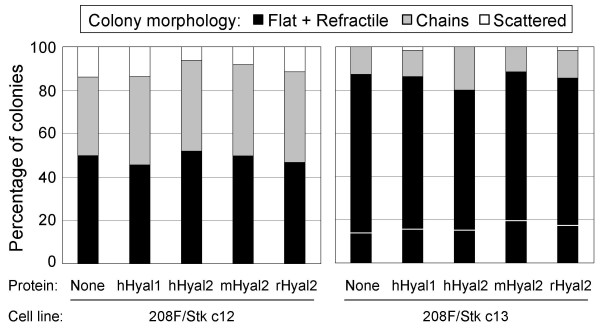
Colony morphology of 208F/Stk cells is unaffected by Hyal2 expression. 208F/Stk c12 and c13 cells were transduced with retrovirus vectors expressing the indicated proteins or were not transduced (None), the cells were seeded at low density, and colony morphologies were evaluated 1 week after seeding. In the right panel, the numbers of flat (bottom black bar) and refractile (top black bar) colonies were counted separately. In the left panel these categories were combined. The experiment was repeated with similar results.

Next we exposed the 208F/Stk c12 and c13 clonal cell lines to retroviral vectors encoding mouse, rat or human Hyal2, or human Hyal1, grew these cells in G418 to select for cells expressing the vector, and quantitated the morphologies of colonies that grew after plating the cells at low density (Fig. 4). There were no consistent changes in morphology in response to any of the Hyal2 proteins by comparison to cells expressing Hyal1 or no additional protein. To test for expression of the Hyal2 proteins in these cells, we exposed the cells to JSRV-pseudotype LAPSN vector and measured the transduction rate (Table 1). As expected based on previous results [15], 208F/Stk cells expressing no additional protein or expressing human Hyal1 were not transduced, cells expressing human Hyal2 were transduced efficiently, cells expressing rat Hyal2 were transduced at about 1/3 the rate of cells expressing human Hyal2, and cells expressing mouse Hyal2 were not transduced. Acquisition of JSRV vector susceptibility by the normally resistant rat 208F cells following transfer of the human and rat Hyal2 vectors indicates that the Hyal2 genes are expressed in functional forms. We cannot conclude from this assay that mouse Hyal2 is expressed in cells transduced with the mouse Hyal2 vector, but the integrated vectors in both packaging lines that were used to make the mouse Hyal2 vectors were sequenced and found to have the correct mouse Hyal2 sequence, so it is likely that normal mouse Hyal2 protein was expressed in the transduced 208F/Stk cells. Together these results indicate that the Hyal2 proteins were expressed in the transduced 208F/Stk cells and argue against the hypothesis that Hyal2 can inhibit the activity of Stk.

### Hyal2 overexpression in RON- or Stk-expressing 208F cells does not affect their response to MSP

To quantitate cellular responses to MSP, cells seeded two days earlier were exposed to MSP at concentrations from 1.2 to 400 ng/ml (in half-log intervals) and the next day the cell morphology was examined. We measured the minimum MSP concentration at which an alteration in morphology was observed and the MSP concentration above which no further morphologic changes were observed. The right panels of Fig. 1 show an example of such extremes of morphology.

The original studies showing an interaction between RON/Stk and Hyal2 studied the human proteins [5], so we first examined the effects of human Hyal2 on the MSP response of 208F/RON cells. Identical responses to MSP were observed for 208F/RON c9 and 208F/RON c10 cells that expressed human Hyal2, human Hyal1, or AP. The responses were first noticeable at 4 ng/ml MSP and were maximal at 120 ng/ml MSP. These results indicate that Hyal2 does not modulate the activation of RON in response to MSP.

We next explored the possible effects of Hyal2 on the MSP response of 208F/Stk cells. We tested human, mouse, and rat Hyal2 proteins to address the possibility that only the rodent Hyal2 proteins would interact with Stk. Identical responses to MSP were observed for 208F/Stk c12 cells that expressed mouse, rat, or human Hyal2, human Hyal1, or no protein. In two independent experiments, the first effects were observed at 1.2 ng/ml MSP and the maximum effect was observed at 12 ng/ml. Identical responses were also observed for 208F/Stk c13 cells that expressed mouse, rat, or human Hyal2, human Hyal1, or no protein. In this case, the responses were first noticeable at 4 ng/ml MSP and were maximal at 40 ng/ml MSP. Untreated 208F/Stk c12 cells are more refractile and express more Stk than the 208F/Stk c13 cells (data not shown), and the higher level and basal activity of Stk in these cells likely explains their higher sensitivity to MSP.

## Discussion

The ability of JSRV Env to transform cells in culture, the identification of Hyal2 as the cell-surface receptor for the Env protein of JSRV, and the localization of Hyal2 to the chromosome 3p21.3 lung cancer tumor suppressor locus suggested the hypothesis that JSRV might cause cancer by inhibiting a tumor suppressor activity of Hyal2 [3]. Support for this hypothesis was provided by transformation studies in the human bronchial epithelial cell line BEAS-2B, which indicated that Hyal2 could suppress the transforming activity of the RON tyrosine kinase, and that JSRV Env expression caused Hyal2 degradation and RON activation [5]. The fact that a dominant-negative kinase-dead version of RON could block transformation by JSRV Env in BEAS-2B cells indicated that the RON pathway was required for transformation.

Unfortunately and despite numerous attempts we have been unable to reliably measure transformation in BEAS-2B cells using the originally described expression plasmids and transfection techniques [5]. Occasionally we observe transformed foci following transfection of the JSRV Env expression plasmid, but often cells transfected with a control non-transforming plasmid exhibit similar foci. We are able to obtain reproducible and obvious transformation of BEAS-2B cells by transducing the cells with a retroviral vector encoding JSRV Env (LJeSN vector, ref. [6]), so presumably the lack of reproducibility is due to poor Env expression following transfection. To avoid these difficulties we pursued further studies of the RON pathway in rodent fibroblast cell lines that have traditionally been used in transformation assays.

Based on earlier findings that Stk could transform NIH 3T3 mouse fibroblasts [23,24], we attempted to isolate cells expressing RON or Stk by isolating transformed foci of cells following transfection of NIH 3T3 cells. However, we were unable to detect transformation of NIH 3T3 cells by RON or Stk expression plasmids, consistent with results from other groups for RON [30-32] and for Stk (Kazuo Nishigaki and Sandra Ruscetti, personal communication). Furthermore, we were unable to repeat the studies showing transformation of NIH 3T3 cells by Stk when we used the same Stk expression plasmid used in the earlier reports [23,24]. These reports showed a very high background transformation rate in the NIH 3T3 cells (50 foci per μg control DNA, ref. [24]; see photographs of cell culture dishes in Fig. 2 of ref. [23]) casting doubt on the reliability of the transformation assay. Unless a very flat subclone of NIH 3T3 cells is used in these assays, as we used here, a high and variable background transformation rate can be observed. Even here we observed a level of background morphologic changes in the NIH 3T3 cells such that low levels of transformation would be difficult to detect.

In contrast, the 208F cells showed no background transformation and allowed us to detect a low frequency of transformation by RON and Stk. This low rate of transformation could be due to a requirement for a very high level of RON/Stk expression that occurs only in a small number of cells, or a requirement for mutations that activate the RON/Stk proteins and occur at low frequency. We isolated several foci of Stk-transformed 208F cells and found that these cells expressed Stk protein of the expected size by western blotting and that the cells responded to MSP, indicating that the Stk protein was not constitutively activated by mutations or deletions, and suggesting that the protein is simply overexpressed in these cells. In contrast, cells from the one focus of cells transformed by transfection of the RON gene were unresponsive to MSP and thus might have been the result of activating mutations or deletions in RON. To generate 208F cells expressing RON we transfected RON with a selectable marker and isolated cells showing a weakly transformed phenotype.

We presume that the low level phenotypic alteration observed in 208F/RON and 208F/Stk cells in the absence of MSP addition is due either to low-level constitutive activity of RON/Stk or to RON/Stk activation by low levels of MSP made by the cells or present in the culture medium. Given that the parental 208F cells show no response to MSP, we conclude that the cell scattering phenotype observed after addition of MSP is a direct measure of RON/Stk activation. The transformed phenotype of these cells in the absence of added MSP and the scattering phenotype observed after treatment of the cells with MSP seem to represent variations of the same transformation phenotype. Indeed, 208F cells transformed by other oncogenes, including JSRV Env, exhibit a scattered phenotype similar to MSP treated 208F cells expressing RON or Stk. Furthermore, 208F cells expressing RON or Stk can exhibit a scattered phenotype in the absence of added MSP, especially if the cells are selected for a highly transformed phenotype by passaging substrate-independent cells that float in the culture medium. Given this interpretation, the response of the RON/Stk-expressing cells to MSP can be considered a form of ligand-dependent transformation, similar to what has been observed previously for cells expressing the receptors for human colony-stimulating factor 1 [33], insulin-like growth factor I [34], granulocyte/macrophage colony-stimulating factor [35], and a hybrid EGF receptor/Xmrk tyrosine kinase [36].

Alternatively, it may be that the basal and MSP-induced phenotypes of the RON/Stk-expressing cells are mediated by separate signaling pathways, as has been found for the related receptor tyrosine kinase MET [37]. These investigators found that a MET oncogene could induce transformed foci and an invasion phenotype in cultured rat fibroblasts, which when injected into nude mice would form tumors and would metastasize to the lungs. Two point mutations in the MET oncogene completely abrogated the invasion and metastasis phenotypes but did not affect the transformation and tumorigenesis phenotypes of the MET oncogene, indicating that separable signaling pathways were involved.

We did not find any effect of human Hyal2 on the basal phenotype or the response to MSP of the 208F/RON cells, nor did we find an effect of human, rat, or mouse Hyal2 on the basal phenotype or the MSP response of 208F/Stk cells. These results argue against direct regulation of RON activity by Hyal2 as was indicated by prior experiments using BEAS-2B cells [5]. It is possible that additional factors present in BEAS-2B but not in 208F cells are required for regulation of RON activity by Hyal2.

Recently we have found that Env proteins from JSRV and from the related retrovirus enzootic nasal tumor virus (ENTV) can transform Madin-Darby canine kidney (MDCK) epithelial cells, but by a mechanism different from that observed in BEAS-2B cells and similar to that observed in rodent fibroblast cell lines [38]. In particular, the cytoplasmic tail of Env is required for transformation, the PI3K/Akt pathway is activated and inhibition of PI3K activity reverses the transformed phenotype, expression of RON (which is not normally expressed in MDCK cells) does not affect transformation, and canine Hyal2 expressed by these cells appears uninvolved. These results show that the JSRV Env protein can transform epithelial cells besides BEAS-2B cells, and argue against a model for Env transformation involving different pathways that are uniquely active in epithelial cells as compared to fibroblasts. Future work will focus on the transformation pathways that are active in lung tumors induced in animals by JSRV Env. While it is possible that the RON pathway plays a role in oncogenesis, most of the evidence points to a main role for the cytoplasmic domain of Env and activation of the PI3K/Akt pathway.

## Conclusions

We have shown that expression of the mouse or human orthologs of the receptor tyrosine kinase Mst1r (called RON in humans and Stk in rodents) can induce phenotypic changes indicative of transformation in a rat fibroblast cell line. These changes are enhanced by treatment of the cells with MSP, the natural ligand for RON/Stk, which induces a pronounced scattering/transformation response. Transformation of the BEAS-2B immortalized human epithelial cell line by JSRV appears to require RON activation, as a result of JSRV Env binding to and degradation of the virus receptor Hyal2, which appeared to negatively regulate RON activity. However, we find no evidence for direct regulation of the basal or MSP-induced RON/Stk activity by Hyal2 in rat fibroblasts.

## Competing interests

None declared.

## Authors' contributions

ADM designed the study, performed most of the experiments, and drafted the manuscript. NSVH and SLL performed the western blot and FACS experiments and provided intellectual input, and SLL performed the preliminary studies on BEAS-2B cells. All authors read and approved the final manuscript.

## Pre-publication history

The pre-publication history for this paper can be accessed here:



## Figures and Tables

**Table 1:** Assay for functional expression of Hyal2 proteins in 208F/Stk cells transduced with Hyal2-expressing retroviral vectors

Source of vector used to transduce cells	LAPSN(PJ4) vector titer (AP^+ ^FFU/ml) on cells:	
	
	208F/Stk c12	208F/Stk c13
No vector	<10	<10
PT67/LhHyal1SN c16	<10	<10
PT67/LhHyal2SN c16	2,000	2,000
PT67/LrHyal2SN c2	800	600
PT67/LrHyal2SN c12	600	600
PT67/LmHyal2SN c7	<10	<10
PT67/LmHyal2SN c8	<10	<10

208F/Stk c12 and 208F/Stk c13 cells were exposed to vectors from the indicated vector-producing packaging cell lines and were exposed to G418 to select for expression of the vector. The cells were then exposed to JSRV-pseudotype LAPSN vector from PJ4/LAPSN cells and the apparent titers were determined.
